# Icotinib inhibits the invasion of Tca8113 cells via downregulation of nuclear factor κB-mediated matrix metalloproteinase expression

**DOI:** 10.3892/ol.2014.2311

**Published:** 2014-07-04

**Authors:** CAILING YANG, JIANGUO YAN, GUOYAN YUAN, YINGHUA ZHANG, DERONG LU, MINGXIN REN, WEIGANG CUI

**Affiliations:** 1 Department of Oral and Maxillofacial Surgery, The First Affiliated Hospital, Xinxiang Medical University, Weihui, Henan 453100, P.R. China; 2 Department of Human Anatomy, Xinxiang Medical University, Xinxiang, Henan 453003, P.R. China; 3 Department of Neurosurgery, The First Affiliated Hospital, Xinxiang Medical University, Xinxiang, Henan 453003, P.R. China; 4 Department of Internal Digestive Medicine, The Third Affiliated Hospital of Xinxiang Medical University, Xinxiang, Henan 453003, P.R. China

**Keywords:** Tca8113 cells, tumor invasion, nuclear factor κB, icotinib, matrix metallopeptidase

## Abstract

Icotinib is an epidermal growth factor receptor tyrosine kinase inhibitor, which has been revealed to inhibit proliferation in tumor cells. However, the effect of icotinib on cancer cell metastasis remains to be explained. This study examines the effect of icotinib on the migration and invasion of squamous cells of tongue carcinoma (Tca8113 cells) *in vitro*. The results of the Boyden chamber invasion assay demonstrated that icotinib reduced cell invasion, suppressed the protein levels of matrix metalloproteinases (MMPs), MMP-2 and MMP-9, and increased the expression of tissue inhibitor of metalloproteinase-1. In addition, icotinib was found to significantly decrease the protein levels of nuclear factor κB (NF-κB) p65, which suggested that icotinib inhibits NF-κB activity. Furthermore, treatment with the NF-κB inhibitor, pyrrolidine dithiocarbamate, suppressed cell invasion and MMP-2 expression. These results suggested that icotinib inhibits the invasion of Tca8113 cells by downregulating MMP via the inactivation of the NF-κB signaling pathways.

## Introduction

Studies have shown that the matrix metalloproteinases (MMPs), including MMP-2 and MMP-9, are key proteases in the invasion and metastasis of human oral squamous carcinoma and breast cancer cells (1,2). The levels of MMPs are regulated by tissue inhibitors of metalloproteinases (TIMPs). TIMP-1 and TIMP-2 have the ability to inhibit tumor invasion and metastasis (3). Therefore, numerous studies have focused on MMPs and their regulatory pathways, to explore the molecular mechanisms for preventing cancer metastasis.

Increased activity of the nuclear factor κB (NF-κB) pathway has been observed in various signaling cascades, including the apoptosis and metastasis cascades (4,5). It has also been reported that follicle-stimulating hormone affects the proliferation and invasion of ovarian cancer cells by regulating the NF-κB signaling pathway (6). Inhibition of the NF-κB pathway may potentially prevent proliferation and invasion in a broader range of tumors. In addition, the NF-κB signaling pathways are significant in regulating the expression of MMPs. The stilbenoid, piceatannol, suppresses breast cancer cell invasion through the inhibition of NF-κB pathways (7). Thus, the NF-κB pathways are potential targets for the treatment of tumors.

The epidermal growth factor receptor (EGFR) has been observed to be upregulated in a number of tumors and is considered a target for cancer therapy (8). EGFR appears to be one of the most promising and effective targets in the treatment of head and neck (9) and breast (10) cancer. Icotinib hydrochloride, a novel and potent selective EGFR tyrosine kinase inhibitor (TKI), has been used in the treatment of patients with non-small cell lung cancer (NSCLC), and exhibits promising efficacy and safety (11,12). In previous studies, icotinib has shown an effective role in reducing proliferation and increasing apoptosis in HCC827 cells (12), and has exhibited antitumor activity in the A431 cell line (13). A recent study also revealed that the toxicity of icotinib was typically low in patients with advanced NSCLC (14), where icotinib was generally well tolerated and exhibited antitumor activity (15).

Although the inhibitory effect of icotinib in the growth of cancer cells is known, the role of icotinib in the inhibition of tumor invasion and metastasis remains unclear. The present study aimed to determine the inhibitory effect of icotinib on metastasis in human tongue carcinoma Tca8113 cells, and its possible underlying mechanisms of action.

## Materials and methods

### Reagents and antibodies

Icotinib was provided by Zhejiang Beta Pharma Co., Inc. (Yuhang, China) and dissolved in 5% dimethyl sulfoxide (DMSO). Rabbit monoclonal anti-human MMP2, MMP9, TIMP1 and TIMP2 antibodies were purchased from Cell Signaling Technology, Inc. (Danvers, MA, USA), while rabbit polyclonal anti-human NF-κB p65 antibody was purchased from Millipore (Billerica, MA, USA) and mouse monoclonal anti-ox histone H1 was purchased from Millipore (Bedford, MA, USA). Pyrrolidine dithiocarbamate (PDTC) was purchased from Sigma-Aldrich (St. Louis, MO, USA).

### Cell culture

The human tongue carcinoma Tca8113 cell line was obtained from the Shanghai Institute of Biochemistry and Cell Biology (Shanghai, China). The Tca8113 cells were cultured in RPMI-1640 medium (Gibco-BRL, Grand Island, NY, USA) supplemented with 10% fetal bovine serum (FBS; Gibco-BRL) and 1% streptomycin (Sigma-Aldrich), and incubated at 37°C in a 5% CO_2_ humidified atmosphere. The medium was replaced three times per week.

### Western blot analysis

Equal amounts of protein lysates were separated on 10% Bis-Tris gels (Invitrogen Life Technologies, Carlsbad, CA, USA) and electrophoretically transferred to 0.22 μm polyvinylidene difluoride membranes (Invitrogen Life Technologies). The membranes were blocked with 5% non-fat milk for 2 h at room temperature and then incubated with the primary rabbit monoclonal anti-human MMP2, MMP9, TIMP1 and TIMP2 (Cell Signaling, Inc.), and rabbit polyclonal anti-human NF-κB p65 (Millipore) antibodies. Following washing with phosphate-buffered saline, the membranes were incubated with horseradish peroxidase-conjugated IgG (Pierce Biotechnology, Inc., Rockford, IL, USA) and then washed a further three times with Tris-buffered saline with Tween 20. The immunoreactivity was visualized by enhanced chemiluminescence (WP20005, Invitrogen Life Technologies). β-actin or His H1 was used as an experimental control, and the density of immunoblotting was quantified using Quantity One software (Bio-Rad, Hercules, CA, USA).

### Boyden chamber invasion assay

The ability of Tca8113 cells to pass through the Matrigel-coated polycarbonate filters (Becton-Dickinson, Heidelberg, Germany) was determined by the Boyden chamber invasion assay. Following treatment with various concentrations of icotinib for 24 h, the cells (1×10^4^ cells/well) in serum-free medium were added to the upper chamber. The complete medium containing 10% FBS was applied to the lower chamber as a chemoattractive agent, and the chamber was subsequently incubated for 24 h at 37°C. Following incubation, the cells of the upper surface of the membrane were removed with a cotton swab. The cells that had invaded across the Matrigel to the lower surface of the membrane were stained with hematoxylin and eosin. These cells were then scored by ImageJ quantification software (National Institutes of Health, Bethesda, MD, USA) under a microscope (DM6000B, Leica Microsystems, Wetzlar, Germany) (16).

### Nuclear protein extraction

The cytosolic and nuclear extracts were prepared as previously described with slight modifications (17). Briefly, the cells were centrifuged in a homogenization buffer [10 mM Hepes, 10 mM KCl, 0.1 mM EGTA, 0.1 mM EDTA, 0.5 mM phenylmethanesulfonyl fluoride (PMSF) and protease inhibitor cocktail (1:100); Active Motif, Carlsbad, CA, USA] at 13,000 × g for 10 min at 4°C, and the supernatant was collected. For the nuclear protein extracts, the pellets were resuspended in nuclear extraction buffer (20 mM Hepes, 1.5 mM MgCl_2_, 0.4 M NaCl,1 mM EGTA, 1 mM EDTA, 1 mM dithiothreitol, 0.5 mM PMSF and protease inhibitor cocktail (1:100)] for 30 min and centrifuged at 13,000 × g for 20 min at 4°C. The supernatants containing the nuclear protein were collected and stored at −80°C.

## Results

### Icotinib inhibits the proliferation of Tca8113 cells

**Figure 1:**
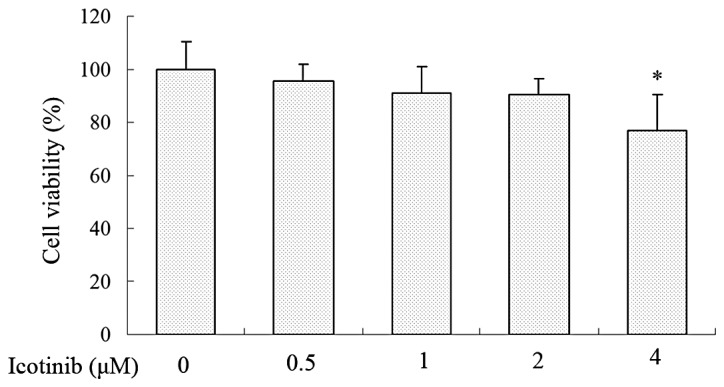
Effects of icotinib on Tca8113 cell viability. MTT assay indicated that treatment with icotinib for 24 h at various concentrations (0 and 2 μM) exhibited no cytotoxicity on Tca8113 cells. Data are presented as the mean ± standard error of the mean (n=5) for each group. ^*^P<0.05, vs. the control group.

Icotinib has been observed to inhibit tumor cell proliferation (18). In the present study, the antiproliferative effects of icotinib at various concentrations (0, 0.5, 1, 2 and 4 μM) on Tca8113 cells for 24 h were determined by MTT assay, and the results are shown in Fig. 1. The viability of Tca8113 cells was not significantly affected by ≤2 μM icotinib compared with the DMSO-treated control group. As a result, a concentration ranging between 0.5 and 2 μM was selected for the subsequent experiments with icotinib.

### Icotinib inhibits the invasion of Tca8113 cells

**Figure 2:**
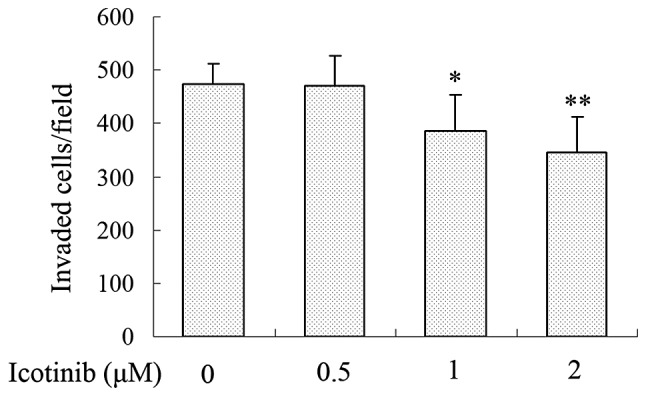
Effect of icotinib on the invasion of Tca8113 cells. The invaded Tca8113 cells were counted in five random fields following treatment with various concentrations of icotinib (0–2 μM) for 24 h. Data are presented as the mean ± standard error of the mean (n=5) for each group. ^*^P<0.05 and ^**^P<0.01, vs. the control group.

To investigate the effect of icotinib on the invasive ability of Tca8113 cells, the motility of Tca8113 cells was examined by Transwell invasion assay. Tca8113 cells treated with icotinib at various concentrations (0, 0.5, 1 and 2 μM) were plated in the upper chamber for 24 h. After 24 h, the number of cells that had moved to the lower membrane was counted under a light microscope (Leica Microsystems). The results demonstrated that icotinib reduced the invasion of Tca8113 cells in a concentration-dependent manner when treated with 0, 0.5, 1 and 2 μM of icotinib for 24 h (Fig. 2).

### Icotinib downregulates the expression of MMP-2 and MMP-9 and promotes the expression of TIMP-1 and TIMP-2 in Tca8113 cells

**Figure 3:**
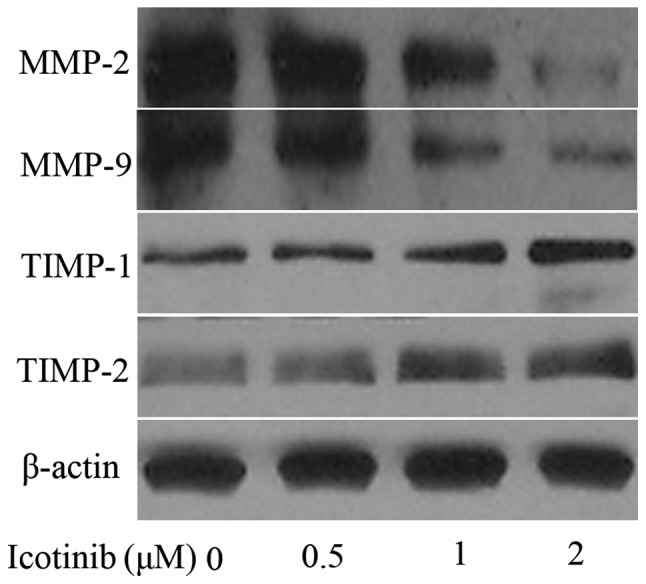
Effects of icotinib on MMP-2, MMP-9, TIMP-1 and TIMP-2 expression. The expression of these proteins was analyzed in cells following treatment with various concentrations of icotinib (0–2 μM) for 24 h. MMP, matrix metalloproteinase; TIMP, tissue inhibitor of metalloproteinases.

As MMP-2 and MMP-9 have a critical function in tumor cell invasion, the inhibitory effect of icotinib on the expression of MMP-2 and MMP-9 was investigated. Tca8113 cells were treated with 0, 0.5, 1 and 2 μM icotinib for 24 h. As shown in Fig. 3, icotinib markedly reduced MMP-2 and MMP-9 expression in a concentration-dependent manner. Additionally, icotinib significantly increased the protein levels of TIMP-1 and TIMP-2 in a concentration-dependent manner, as shown in Fig. 3 by western blot analysis.

### NF-κB pathway is involved in the antimetastatic mechanism of icotinib

**Figure 4:**
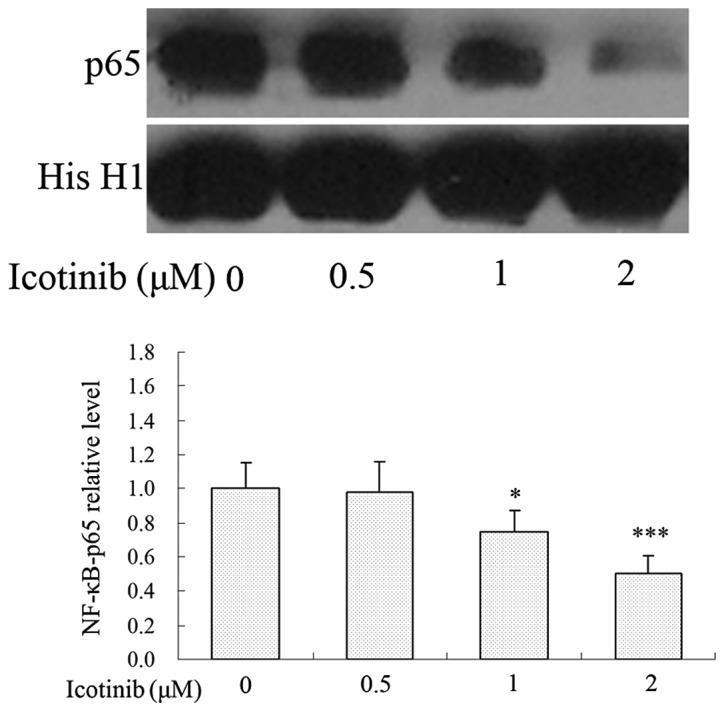
Effects of icotinib on the expression of NF-κB p65. The nuclear extracts were prepared and the protein levels of NF-κB p65 were determined using western blot analysis following treatment with various concentrations of icotinib (0–2 μM) for 24 h. His H1 was used as a loading control and the densitometry was quantified. Data are presented as the mean ± standard error of the mean (n=5) for each group. ^*^P<0.05 and ^***^P<0.001, vs. the control group. NF-κB, nuclear factor κB.

**Figure 5:**
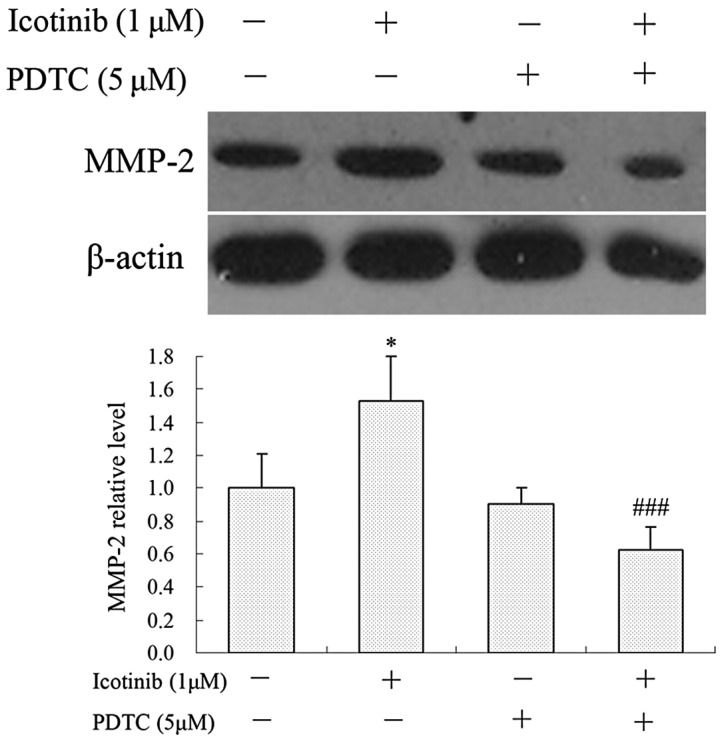
Effects of the nuclear factor κB inhibitor, PDTC, and icotinib on MMP-2 expression in Tca8113 cells. Cells were pretreated with PDTC (5 μM) for 1 h and then incubated in the presence or absence of icotinib (1 μM) for 24 h. The protein levels of MMP-2 were measured by western blot analysis. β-actin was used as a loading control and the densitometry was quantified. Data are presented as the mean ± standard error of the mean (n=5) for each group. ^*^P<0.05 and ^###^P<0.001, vs. the control group. PDTC, pyrrolidine dithiocarbamate; MMP, matrix metalloproteinase.

To elucidate whether the inhibitory effect of icotinib on the lower expression of MMP-2 is regulated by the NF-κB pathway, the effect of icotinib on the NF-κB pathway in Tca8113 cells was investigated. The results shown in Fig. 4 demonstrate that the nuclear levels of NF-κB p65 were significantly decreased following treatment with icotinib in comparison with the control. To further determine whether the effect of icotinib on lowering the levels of MMP-2 occurred through the inhibition of NF-κB pathway, Tca8113 cells were then pretreated with the NF-κB inhibitor, PDTC (5 μM), for 1 h and incubated in the presence or absence of icotinib (1 μM) for 24 h. The results indicated that PDTC significantly reverses the effect of icotinib on the expression of MMP-2 (Fig. 5).

## Discussion

The antitumor effect of icotinib has been confirmed in a number of cancer cell lines (13,18). However, the anti-invasion effect and its associated mechanisms in Tca8113 cells were unclear. The current study revealed that icotinib significantly suppresses the invasion and metastasis of Tca8113 cells by regulating the expression of MMPs and TIMPs via the inhibition of the NF-κB pathway. To the best of our knowledge, the antimetastatic effects of icotinib *in vitro* have rarely been reported.

The present study demonstrated that icotinib does not inhibit the viability of Tca8113 cells treated with icotinib at non-toxic doses; however, the cell invasion was inhibited. These results suggested that the invasion-inhibiting effect of icotinib on Tca8113 cells was not due to its cytotoxicity. To clarify the mechanism of icotinib in the inhibition of invasion, the potential correlation between the inhibitory effect of icotinib on cell invasion and the downregulation of MMP levels were investigated

Cancer cell invasion and metastasis are the predominant causes of mortality in cancer patients. MMPs are significant in degrading the matrix barriers around the tumor, and contribute to cell invasion and metastasis (7,19). TIMPs are inhibitors of MMPs, which control MMP activities and minimize matrix degradation (20,21). To determine whether the inhibitory effect of icotinib on cell invasion and metastasis is associated with MMPs in Tca8113 cells, a Boyden chamber invasion assay was performed with icotinib-treated cells. Icotinib markedly inhibited the expression of MMPs, while the levels of TIMP-1 and TIMP-2 were increased following icotinib treatment in Tca8113. The results presented in this study suggested that icotinib suppresses tumor cell invasion by downregulating MMPs and upregulating TIMP-1/2.

Several studies have revealed that NF-κB activation is involved in stimulating the secretion of MMPs in tumor cells (22,23). A previous study has also demonstrated that the steroid saponin, diosgenin, inhibits tumor necrosis factor-induced NF-κB activation and blocks the proliferation of tumor cells (24). Furthermore, when activated, NF-κB has been shown to stimulate invasion and metastasis in a number of cancer cell lines, which mediates the resistance to chemo- and radiotherapies (2,25). The current study demonstrated that icotinib suppresses the protein expression of NF-κB p65, and that treatment with the NF-κB inhibitor, PDTC, significantly reduces the cell invasion and decreases the levels of MMP. These results demonstrated that the reduction in MMP expression levels is caused by icotinib and attributed to the blocking of NF-κB activation.

In conclusion, the results of this study demonstrated that icotinib has the ability to inhibit the migration and invasion of the squamous cells of tongue carcinoma *in vitro*. This effect may be via the inactivation of NF-κB-mediated MMP-9 expression. Therefore, icotinib may present as a promising antimetastatic drug for the prevention of malignant cancer.
